# The Role of Salvage Radical Prostatectomy in Patients with Radiation-Resistant Prostate Cancer

**DOI:** 10.3390/cancers15143734

**Published:** 2023-07-23

**Authors:** Jake Drobner, Alain Kaldany, Mihir S. Shah, Saum Ghodoussipour

**Affiliations:** 1 Division of Urologic Oncology, Rutgers Cancer Institute of New Jersey and Rutgers Robert Wood Johnson Medical School, New Brunswick, NJ 08901, USA; 2 Department of Urology, Sidney Kimmel Cancer Center, Thomas Jefferson University, Philadelphia, PA 19107, USA

**Keywords:** prostate cancer, salvage therapy, salvage prostatectomy, post-radiation prostatectomy, post-radiation recurrence, biochemical recurrence, radiation-resistant prostate cancer

## Abstract

**Simple Summary:**

Prostate cancer affects one out of every eight men over the course of their lifetimes, and recurrence of the disease after initial cancer treatment occurs in almost one-third of men. The aim of our review was to assess the role of salvage radical prostatectomy in patients with prostate cancer recurrence after initial treatment with radiotherapy. Outcome data demonstrate that radical prostatectomy has oncologic benefits, although it incurs several intraoperative and postoperative functional risks including rectal injury, erectile dysfunction, and incontinence. Given that there is no clinical consensus on which management approach is superior for patients with localized prostate cancer recurrence, this review provides evidence that radical prostatectomy offers meaningful cancer control and should be considered for select patients with radiation-resistant disease.

**Abstract:**

There are multiple treatment strategies for patients with localized prostate adenocarcinoma. In intermediate- and high-risk patients, external beam radiation therapy demonstrates effective long-term cancer control rates comparable to radical prostatectomy. In patients who opt for initial radiotherapy but have a local recurrence of their cancer, there is no unanimity on the optimal salvage approach. The lack of randomized trials comparing surgery to other local salvage therapy or observation makes it difficult to ascertain the ideal management. A narrative review of existing prospective and retrospective data related to salvage radical prostatectomy after radiation therapy was undertaken. Based on retrospective and prospective data, post-radiation salvage radical prostatectomy confers oncologic benefits, with overall survival ranging from 84 to 95% at 5 years and from 52 to 77% at 10 years. Functional morbidity after salvage prostatectomy remains high, with rates of post-surgical incontinence and erectile dysfunction ranging from 21 to 93% and 28 to 100%, respectively. Factors associated with poor outcomes after post-radiation salvage prostatectomy include preoperative PSA, the Gleason score, post-prostatectomy staging, and nodal involvement. Salvage radical prostatectomy represents an effective treatment option for patients with biochemical recurrence after radiotherapy, although careful patient selection is important to optimize oncologic and functional outcomes.

## 1. Introduction

Prostate cancer remains the most common non-dermatologic cancer among men in the United States [1]. With an estimated 268,490 new cases and 34,500 deaths in 2022 alone, prostate cancer will affect 1 out of every 8 men over the course of their lifetimes [1]. Despite the prevalence and public health implications of prostate cancer, guidelines for screening using prostate-specific antigen (PSA) remain variable among physician organizations [2]. As of 2023, the American Urological Association (AUA) recommends a shared decision-making model between clinician and patient, with PSA screenings every 2–4 years initiated at 45–50 years of age for patients with average risk [3]. Fortunately, greater than 70% of prostate cancer is diagnosed at a localized stage, and the five-year survival rate for prostate cancer is the highest of all cancers at nearly 100% [1]. For localized prostate adenocarcinoma, the National Comprehensive Cancer Network (NCCN) recommends treatment strategies by stratifying patients into six risk categories based on a digital rectal examination, serum PSA, a prostate biopsy, and imaging studies. The primary treatment strategies endorsed—which are consistent with recommendations from the AUA and the American Society of Therapeutic Radiology and Oncology (ASTRO) [4]—are active surveillance, external beam radiation therapy (EBRT) with or without androgen deprivation therapy (ADT), brachytherapy, or open or robot-assisted laparoscopic radical prostatectomy (RP) [5]. Still, while these treatment modalities offer excellent and comparable long-term survival, a subset of patients experience disease recurrence despite primary treatment [6].

The serial evaluation of serum PSA is the primary surveillance method in males who have undergone initial treatment for localized prostate cancer. Despite undergoing definitive treatment for localized prostate cancer, an estimated 20–50% of men will experience rising PSA levels with no clinical evidence of disease relapse [7]—also known as biochemical recurrence (BCR). The AUA defines BCR as a post-prostatectomy serum PSA ≥ 0.2 ng/mL, followed by a subsequent confirmatory serum PSA value ≥ 0.2 ng/mL [8]. ASTRO’s Phoenix criteria define BCR following EBRT specifically; the criteria state that a serum PSA rise of ≥2 ng/mL above the nadir PSA is considered BCR after EBRT, regardless of whether the patient received ADT [9]. Twenty-seven percent of BCR occurs within five years of initial treatment, but the risk of BCR does not plateau for at least 15 years [10]. Imaging is critical for the localization of the recurrence, which enables providers to accurately restage patients and therefore determine the most appropriate salvage therapy regimen. Positron emission tomography (PET) using novel radiotracers that target prostate-specific membrane antigen (PSMA) has demonstrated increased sensitivity and specificity for detecting micrometastatic disease at both initial staging and at BCR, especially in patients with lower PSA values [11,12,13]. In patients with concern for recurrence, multiparametric magnetic resonance imaging (mpMRI) of the prostate is a well-validated tool and the imaging modality of choice for previously irradiated patients. mpMRI can identify residual disease in the irradiated prostate as well as any locoregional recurrences, including extraprostatic extension and seminal vesicle invasion [14,15,16]. mpMRI is also useful to guide confirmatory prostate biopsy. Because negative prostate re-biopsy rates after irradiation range from 62 to 80%, a consensus statement by ASTRO recommends that prostate re-biopsy should only be performed if salvage therapy is being planned [17].

Among men with biopsy-proven post-radiation recurrence, there is currently no consensus on the optimal approach for salvage therapy [18]. Current options include RP, EBRT, brachytherapy, focal therapies, ADT, and enrollment in clinical trials, but there is a dearth of randomized clinical trials comparing these different options with each other or with observation. In fact, in a meta-analysis of 150 studies of local salvage therapies after radiotherapy for prostate cancer, adjusted recurrence-free survival rates were equivocal across modalities at 5 years [19]. Regardless of salvage therapy choice, there is a clinical imperative to treat men who experience BCR after radiation therapy (RT) because these men are at an increased risk of developing metastatic disease and dying of prostate cancer [20]. In combination with the patient’s age and overall health, clinical and pathologic parameters at the time of original diagnosis are helpful in predicting the patients who are most likely to benefit from intervention. Men with pre-salvage PSA levels > 10 ng/mL, pre-salvage T3/T4 disease, or pre-salvage Gleason scores ≥ 7 on re-biopsy are unlikely to be cured by local salvage therapy [21]. The objective of this review is to assess the efficacy, complications, and outcome data reported on salvage radical prostatectomy in patients with prostate cancer recurrence after radiotherapy.

## 2. Methods

Systematic literature searches in MEDLINE (via Pubmed), Google Scholar, and clinicaltrials.gov databases were undertaken to identify published materials on oncologic and functional outcomes of salvage RP after primary RT for prostate cancer. All articles available in English and published after the year 1993 were considered. Letters, editorials, meeting abstracts, replies from authors, and case reports with fewer than 10 patients were excluded. Given that there is no strong evidence or clinical consensus on which of the several therapeutic options for patients with localized post-radiation recurrence is superior, this review will highlight the advantages and discuss the disadvantages of surgical intervention in previously irradiated patients based on surgical approach and primary radiotherapy type.

## 3. Results and Discussion

**Figure 1:**
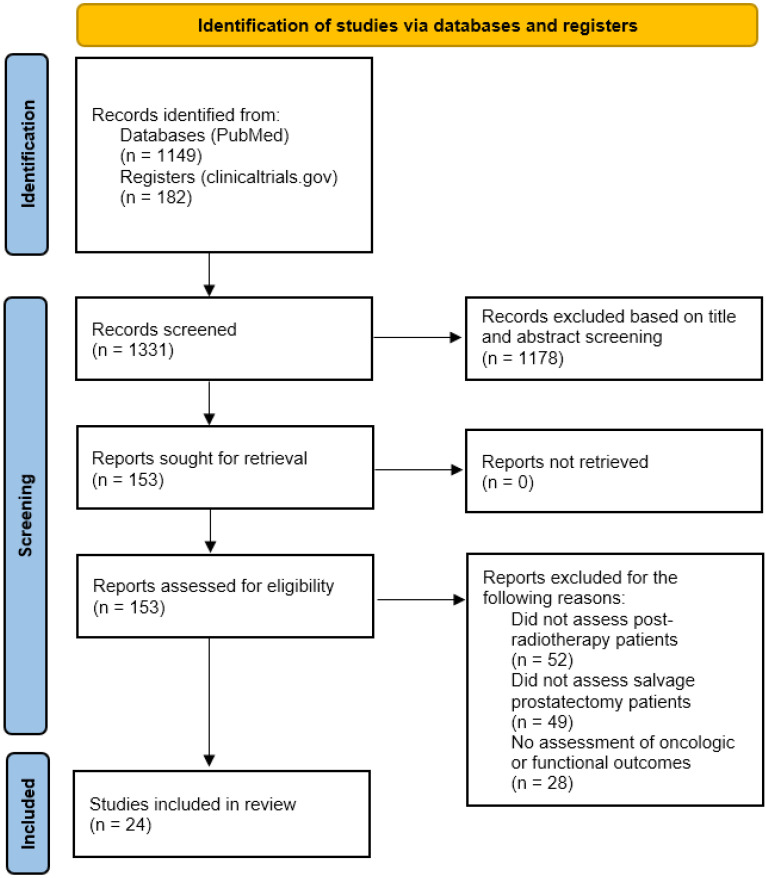
Flow diagram for study selection according to PRISMA 2020 guidelines.

Our search identified 1331 studies for consideration, including 182 registered clinical trials. After exclusion and screening, 24 total studies were included in our review, including three clinical trials identified via the clinicaltrials.gov register. Flow diagram for study selection according to PRISMA 2020 guidelines is shown in Figure 1 [22].

### 3.1. Oncologic Outcomes

Although no randomized clinical trial data for salvage radical prostatectomy after radiotherapy exist, several multi- and single-center studies have reported both retrospective and prospective oncologic outcomes in this population (Table 1). The studies with the largest patient populations were multicenter, retrospective studies. The reported survival metrics varied across studies, with all studies reporting either BCR-free survival (BFS) or progression-free survival (PFS) as the primary outcome for quantifying recurrence. Recurrence was defined in most studies as a PSA rise > 0.2 ng/mL, although some studies used a higher threshold of >0.4 ng/mL. Some studies additionally reported cancer-specific survival (CSS), metastasis-free survival (MFS), and overall survival (OS). There was a wide range of follow-up times across the examined studies, with most studies (88%) reporting survival statistics for at least 5 years after surgery. Only 35% of studies reported survival statistics for 10 years after surgery. Overall, 5-year BFS/PFS ranged from 39 to 61% and 10-year BFS/PFS ranged from 31 to 48%. MFS was only reported in three studies, ranging from 75 to 90% at 5 years and from 65 to 77% at 10 years. CSS ranged from 89 to 95% at 5 years and from 65 to 83% at 10 years. OS ranged from 84 to 95% at 5 years and from 52 to 77% at 10 years.

In one of the earliest studies on salvage RP after radiotherapy, Rogers et al. examined 40 patients with clinically localized (T1-T3N0) disease. The authors reported 55% PFS at 5 years, and the only factor significantly predictive of progression was a pre-RP serum PSA level greater than 10 ng/mL [23]. Tefilli et al. reported a slightly lower 5-year PFS of 44.4% in a group of 27 patients, noting that all patients with pathologically organ-confined disease were without recurrence at 3 years [24]. Lerner et al. examined a larger sample of 132 patients, although only 37 had T1-T3N0 disease. In this cohort, 10-year BFS was 47.3% after salvage RP, and a significant survival advantage was associated with both negative surgical margins and non-aneuploid tumors; pre-RP serum PSA levels were not analyzed [25]. Gheiler et al. followed 40 patients after salvage RP and reported 47.4% BFS at 3 years. Again, serum PSA > 10 ng/mL was associated with a higher risk of biochemical recurrence, but this was not significant in their study (73.7% vs. 31.6%, *p* = 0.65). Instead, pathologically organ-confined disease was a statistically significant predictor of BFS; seminal vesicle invasion (SVI) and positive lymph nodes were the worst prognosticators [26]. In the largest study with the longest follow-up published before the turn of the millennium, Amling et al. reported a 10-year post-salvage RP PFS of 43% for 108 prostate cancer patients. As in the analysis of Lerner et al., DNA ploidy was the strongest predictor of survival (*p* = 0.0002). Serum PSA > 10 ng/mL also correlated with lower PFS (70% vs. 47%, *p* = 0.057), but the interval between radiotherapy and salvage prostatectomy did not significantly affect PFS [27]. Taken together, these early studies suggest that the group of patients most likely to have durable disease control at 5 and even 10 years after surgery are those with lower pre-salvage serum PSA scores, non-aneuploid tumors, and organ-confined disease.

As concerns around the surgical morbidity associated with salvage radical prostatectomy faded, and urologists became less reluctant to perform salvage RP, results of earlier studies were built upon by more contemporary outcomes in the 21st century. Ward et al. retrospectively analyzed outcomes from 199 patients at a single center and reported an overall PFS of 48% at 10 years. Ploidy demonstrated significant predictive power for PFS (*p* < 0.05), and preoperative PSA > 10 ng/mL was again associated with worse survival (*p* = 0.007). The interval from RT to surgery did not correlate with PFS [28]. In a large, international, multi-institutional analysis of 404 patients, Chade et al. reported a 10-year BFS of 37%. The authors found that pre-RP serum PSA and pathologic Gleason score at RP predicted both recurrence (*p* = 0.014) and metastasis (*p* < 0.001), suggesting that the ideal salvage RP patients have pre-RP PSA < 4 ng/mL and post-radiation biopsy Gleason scores of 7 or less. Notably, freedom from clinical metastasis was observed in 77% of patients 10 years after salvage prostatectomy [29]. Similar studies with shorter follow-up periods corroborated these results (Table 1) [30,31,32,33,34,35]. Overall, these studies confirmed the role of pre-SP serum PSA as a predictive marker and provided stronger evidence on the prognostic value of pre-SP Gleason scores.

Studies published within the last five years provide the most comprehensive data on salvage radical prostatectomy outcomes and underscore improvements in surgical technique as well as novel robotic-assisted approaches. In 2019, Mohler et al. published the results of CALGB 9687 (Alliance), the first prospective, multi-institutional, single-arm salvage RP trial. The authors found that the 10-year BFS was 33% and that the 10-year OS was 52% [36]. These lower rates likely reflect the fact that previous studies were mostly retrospective and followed non-protocol recruitment. However, Mohler et al. reported a lower positive surgical margin rate (17%) relative to Chade et al. (25%) and Sanderson et al. (36%), reflecting improvements in overall surgical experience and skill. In a systematic review of salvage radical prostatectomy after radiotherapy, Grubmüller et al. reported a similar 10-year BFS of 31–37% across 2323 patients [37]. Pre-RP serum PSA, pre-RP biopsy Gleason Score, and pathologic lymph node involvement were the strongest prognostic factors for good outcomes. Grubmüller et al. were one of the first to report on outcomes related to the laparoscopic/robotic-assisted surgical approach, which significantly increased over time (0% vs. 22.4% after 2010, *p* < 0.0001). The authors found no difference in positive surgical margin rates (*p* = 0.13) and the pathologic stage after surgery (*p* = 0.55) when comparing robotic-assisted surgery to open surgery [37]. Catarino et al. examined 29 patients treated with laparoscopic salvage RP and reported that 5-year BFS was 50% with a positive surgical margin rate of 27.6%, although surgical margin status and pre-RP biopsy Gleason score did not affect BFS. However, positive lymph nodes, high pre-RP serum PSA, and high TNM stage were correlated with worse BFS [38]. Most recently, Calleris et al. looked to validate the European Association of Urology (EAU) guidelines that recommend restricting salvage radical prostatectomy for radio-recurrent prostate cancer to a favorable-prognosis group: those with organ-confined prostate cancer ≤ stage T2b, pre-RP Gleason score ≤ 7, and pre-SP PSA levels <10 ng/mL [39]. Of the 1030 men in their study, 221 fully met EAU criteria, and the EAU-compliant group experienced more favorable pathological outcomes; the authors reported improved 5-year MFS (90% vs. 76%, *p* < 0.001), 5-year BFS (55% vs. 38%, *p* < 0.001), and 5-year OS (89% vs. 84%, *p* = 0.01) [40]. Overall, the results from these recent series highlight that salvage radical prostatectomy after radiotherapy can achieve excellent tumor control via careful patient selection based on preoperative serum PSA, Gleason scores, and clinical stage.

**Table 1:** Studies assessing oncologic outcomes for salvage radical prostatectomy after radiation therapy.

Authors (Year)	Study Type and Follow Up Period	N	BFS (95% CI)	PFS (95% CI)	MFS (95% CI)	CSS (95% CI)	OS (95% CI)	Factors Associated with Improved Oncologic Outcomes	Level of Evidence
Grubmuller et al. (2021) [35]	Systematic review, 10 years	2232	31–37%	--	65–72%	72–83%	--	- Lower pre-RP PSA levels - Lower pre-RP Gleason Score - Lower post-RP pathologic stage - No lymph node involvement	Level 2
Mohler et al. (2019) [34]	Prospective, multicenter, 10 years	41	33%	--	--	--	52%	--	Level 2
Chade et al. (2011) [27]	Retrospective, multicenter, 10 years	404	37% (31–43)	--	77% (71–82)	83% (76–88)	77% (71–82)	- Lower pre-RP PSA levels - Lower pre-RP Gleason score	Level 3
Amling et al. (1999) [25]	Retrospective, single-center, 10 years	108	--	43%	--	70%	--	- Pre-RP PSA < 10 - Lower tumor DNA ploidy	Level 3
Lerner et al. (1995) [23]	Retrospective, single-center, 10 years	79	47.3%	--	--	72%	64%	- Non-aneuploid tumors - Negative surgical margins	Level 3
Ward et al. (2005) [26]	Retrospective, single-center, 10 years	199	48%	--	--	65%	--	- Lower tumor ploidy - Lower % 4/5 Gleason grade - Lower pathological stage	Level 3
Calleris et al. (2023) [38]	Retrospective, multicenter, 5 years	1030	38–55%	--	75–90%	--	84–89%	- Pre-RP PSA < 10 - Lower grade on pre-SP biopsy - Pre-RT TMN stage T1 or T2 - No lymph node involvement	Level 3
Catarino et al. (2022) [36]	Prospective, single-center, 5 years	29	50%	--	--	--	--	- Lower pre-RP PSA levels - Lower TNM stage - No lymph node involvement	Level 2
Gorin et al. (2011) [32]	Retrospective, single-center, 5 years	24	39%	--	--	--	90%	- No extracapsular extension	Level 3
Paparel et al. (2009) [31]	Retrospective, single-center, 5 years	146	54% (44–63)	--	--	89%	--	- Lower pre-RP PSA levels - Lower pre-RP Gleason scores	Level 3
Pisters et al. (2009) [30]	Retrospective, single-center, 5 years	42	61%	--	--	--	95%	--	Level 3
Sanderson et al. (2006) [29]	Prospective, multicenter, 5 years	51	--	47% (39–55)	--	--	85% (80–90)	- Pre-RP PSA < 5 - Gleason score < 7	Level 2
Bianco et al. (2005) [28]	Prospective, single-center, 5 years	100	--	55% (46–64)	--	--	--	- Lower pre-RP PSA levels - Lower Gleason scores - No seminal vesicle invasion - No lymph node involvement	Level 2
Tefilli et al. (1998) [22]	Retrospective, single-center, 5 years	27	44.4%	--	--	--	--	--	Level 3
Rogers et al. (1995) [21]	Retrospective, single-center, 5 years	40	--	55% (35–75)	--	95%	--	- Pre-RP PSA level < 10 - No seminal vesicle invasion - No lymph node involvement	Level 3
Yuh et al. (2014) [33]	Prospective, single-center, 3 years	51	57%	--	--	--	--	- Lower pre-RP PSA levels - No extracapsular extension	Level 2
Gheiler et al. (1998) [24]	Retrospective, single-center, 3 years	40	47.4%	--	--	--	--	- Lower pre-RT TMN stage - Organ-confined disease	Level 3

BFS = BCR-free survival; PFS = progression-free survival; MFS = metastasis-free survival; CSS = cancer-specific survival; OS = overall survival.

### 3.2. Functional Outcomes and Complications

The high rates of post-surgical incontinence and erectile dysfunction have been major impediments to the acceptance of salvage radical prostatectomy. Functional morbidity associated with salvage radical prostatectomy is significant but has decreased over time with improvement in surgical techniques (Table 2). For example, only two of the nine studies published on or before 2010 reported 1-year incontinence rates after salvage RP lower than 50%, whereas four out of six studies published after 2010 reported 1-year incontinence rates lower than 50%. However, salvage RP is still a technically challenging operation because radiation induces fibrosis, alters tissue planes, and slows down tissue healing, all of which increase the risk of inadvertent intraoperative injuries and postoperative stricture or anastomotic leak compared to primary RP [41]. Furthermore, many irradiated patients endorse some level of preoperative urinary leakage and most endorse erectile dysfunction, making it difficult to isolate and assess surgery-specific changes [42,43]. In a propensity-matched cohort analysis of 53 patients, Bates et al. found that, compared to patients who underwent salvage radical prostatectomy, those who underwent primary robotic-assisted prostatectomy were more likely to return to both continence (*p* < 0.001) and potency (*p* = 0.043) [41].

Stephenson et al. were the first to illuminate that the functional morbidity associated with salvage RP has declined significantly over time. In a review of 100 cases, the authors found that grade II or greater complication rates had decreased significantly over the twenty-year time period from 1984 to 2003 (33% pre-1993 vs. 13% post-1993, *p* = 0.02), including the rectal injury rate (15% vs. 2%, *p* = 0.01) [44]. Continence rates increased slightly over time from 57% to 68%, but this was not statistically significant. The overall erectile potency rate was 16%, highlighting that erectile dysfunction is likely inevitable [44]. Although it is hard to say which clinical or pathologic factors outside of preoperative continence and erectile function may help to predict postoperative morbidity, Calleris et al. did find that the EAU-compliant group had improved continence rates (79% vs. 63%, *p* < 0.001) [40]. This suggests that organ-confined prostate cancer ≤ stage T2b, a pre-RP Gleason score ≤ 7, and pre-SP PSA levels < 10 ng/mL may be valuable prognosticators for functional outcomes in addition to oncologic outcomes.

The advent of Retzius-sparing robotic-assisted prostatectomy—which historically improves continence rates— led Mason et al. to examine functional outcomes between 45 traditional robotic-assisted cases and 81 Retzius-sparing salvage prostatectomies. Thirty-day complication rates were lower for the Retzius-sparing group (10% vs. 26%), with 0 rectal injuries. Continence outcomes were also significantly improved in the Retzius-sparing group (59% vs. 38%), although erectile impotency was similar across groups [45]. Notably, the rates of positive surgical margins and BCR were not significantly different between the groups, suggesting that a Retzius-sparing approach is safe and feasible for salvage prostatectomy.

In a small but early study of 18 patients who underwent salvage robotic-assisted RP, Eandi et al. reported a relatively low continence rate of 33% but demonstrated that functional outcomes were otherwise comparable to those of open salvage RP. Moreover, there were 0 rectal injuries when using the robotic approach [46]. Other retrospective studies followed suit, specifically examining complication rates between open versus robotic-assisted salvage radical prostatectomy. In demographic- and preoperative-risk-matched cohorts, Kenney et al. found that there was no statistically significant difference in the frequency (70% vs. 78.5%, *p* = 0.785) or severity (30% vs. 15.7% Clavien > 3, *p* = 0.501) of complications between the robotic and open groups at 90 days [47]. Notably, the robotic approach was associated with lower estimated blood loss, and no rectal injuries occurred in the robotic cohort compared to the two that occurred in the open cohort [47]. From a disease control perspective, there was no difference in positive surgical margin rate between the robotic and open cohorts (15% vs. 15.7%, *p* = 0.709). In a larger review comparing open and robotic-assisted approaches for 395 salvage radical prostatectomies, Gontero et al. reported no differences in overall and major complications (*p* = 0.67 and 0.16, respectively) [48]. However, anastomotic strictures (16.57% vs. 7.66%, *p* = 0.0014) and rectal injuries (2.96% vs. 0.48%, *p* = 0.0934) were more common for the open RP group while urine leakage was more common for the robotic RP group (18.66% vs. 8.88%, *p* = 0.0069). Again, the authors found that the robotic group had a lower average blood loss (222 vs. 715 mL, *p* < 0.0001). Ultimately, these studies highlight that, regardless of the operative approach, incontinence remains common and is the greatest disadvantage of undergoing salvage RP. When considering the approach for post-radiation salvage RP, robotic RP seems to incur a lower risk of rectal injury and lower blood loss compared to open, although overall complication rates are statistically similar.

**Table 2:** Studies assessing functional outcomes for salvage radical prostatectomy after radiation therapy.

Authors (Year)	N	Incontinence at 1 Year, %	Anastomotic Stricture	Erectile Dysfunction at 1 Year, %	Rectal Injury	Venous Thromboembolism	Infection	Blood Transfusion	Hospital Length of Stay, Days	Level of Evidence
Calleris et al. (2023) [38]	221	21%	--	--	--	--	--	--	--	Level 3
Catarino et al. (2022) [36]	29	79%	2	85%	2	1	1	1	--	Level 2
Gontero et al. (2019) [46]	395	43%	39	85%	--	--	--	--	--	Level 3
Mohler et al. (2019) [34]	41	40%	14	45%	3	--	5	13	--	Level 2
Kenney et al. (2016) [45]	39	90%	10	--	2	2	15	--	--	Level 3
Yuh et al. (2014) [33]	51	--	8	--	1	2	4	--	--	Level 2
Gorin et al. (2011) [32]	24	35%	4	28%	0	--	--	19	--	Level 3
Eandi et al. (2010) [44]	18	67%	3	100%	0	--	--	--	--	Level 3
Sanderson et al. (2006) [29]	51	40%	21	--	1	--	--	--	--	Level 2
Bianco et al. (2005) [28]	100	68%	--	--	1	--	--	--	--	Level 2
Ward et al. (2005) [26]	138	33%	22	--	9	--	4	86	--	Level 3
Stephenson et al. (2004) [42]	100	61%	30	84%	7	1	1	--	--	Level 2
Amling et al. (1999) [25]	108	50%	23	--	6	6	--	46	--	Level 3
Gheiler et al. (1998) [24]	30	50%	--	--	--	--	--	--	7.7 (2–30)	Level 3
Lerner et al. (1995) [23]	37	93.6%	16	--	5	5	4	--	8 (2–44)	Level 3
Rogers et al. (1995) [21]	40	58%	11	--	6	--	3	2	9.6 (6–16)	Level 3

### 3.3. Additional Considerations

#### 3.3.1. Effect of Primary Therapy on Salvage Prostatectomy Outcomes

As mentioned previously, primary treatment options for localized prostate include radical prostatectomy, radiation therapy, and focal therapy. As such, salvage RP may be offered in multiple scenarios, namely, post-radiation and post-focal therapy, and there is growing evidence that outcomes after salvage RP may differ based on which primary therapy a patient receives. Onol et al. retrospectively evaluated a group of 126 patients who underwent salvage RP following radiation or focal therapy. Postoperatively, those who received primary focal therapy were noted to have significantly higher continence rates compared to those who received primary RT (77.3% vs. 39.2%, *p* = 0.002), while no difference was noted in five-year BFS (59% vs. 56%; *p* = 0.761) [49]. Ribeiro et al. similarly compared outcomes following salvage RP after RT or focal therapy in a retrospective cohort of 185 patients and found that patients who had salvage RP after RT had a higher overall complication rate (34% vs. 5%, *p* < 0.001) and a lower complete continence rate (49% vs. 83% pad-free), whereas potency and 3-year BFS were statistically similar between groups [50]. Together, these studies suggest worsened functional outcomes with similar survival outcomes for patients undergoing salvage RP following primary radiotherapy compared to primary focal therapy, although long-term data are lacking.

#### 3.3.2. The Role of Lymph Node Dissection and Seminal Vesicle Biopsy

While the presence of nodal involvement has been correlated with negative outcomes, the practice of pelvic lymph node dissection (PLND) at time of salvage RP is not universal. In a retrospective study of the Surveillance, Epidemiology, and End Results (SEER) database, PLND was performed in fewer than 30% of patients undergoing salvage RP [51]. Furthermore, those who did undergo PLND were noted to have increased CSS, with the lymph node count showing a direct correlation with increasing CSS up to seven nodes [51]. In a separate retrospective multicenter cohort, pathologic nodal involvement was found to be associated with an increased risk of BCR, the development of metastasis, and decreased OS among patients undergoing salvage RP for radio-recurrent prostate cancer [52]. Given these prognostic and potential therapeutic benefits, performing PLND at the time of salvage RP should be standard of practice when possible.

The presence of seminal vesicle invasion may also be a relevant prognosticator for patients undergoing salvage radical prostatectomy after radiation therapy. The rate of SVI is approximately 7% in high-risk prostate cancer, and SVI has been associated with an increased risk of BCR and worse survival [53,54]. In men who initially receive radiotherapy for localized prostate cancer, the seminal vesicles receive lower doses of EBRT to avoid rectal exposure and are often not targeted by brachytherapy [55]. A study by Meeks et al. of 206 men who underwent salvage RP after radiation found that SVI was detected in 32% of patients postoperatively; there was no difference in SVI across patients treated with EBRT vs. brachytherapy. However, 5-year BFS was significantly higher in patients without SVI (56% vs. 18%, *p* < 0.001), and 5-year OS was also higher in men without SVI (98% vs. 94%, *p* = 0.003) [56]. This suggests that there may be prognostic utility to assessing recurrent prostate cancer with seminal vesicle biopsy after radiation. The sensitivity, specificity, positive predictive value, and negative predictive value of seminal vesicle biopsy have been reported as 83%, 100%, 100%, and 84%, respectively [57]. Thus, a post-radiation seminal vesicle biopsy can be critical for diagnosing SVI early and promptly proceeding with salvage radical prostatectomy rather than continuing to surveil, especially if post-radiation serum PSA levels are >10 ng/mL [58].

#### 3.3.3. Novel Biomarkers

One recent study explored the role of a novel biomarker commonly ordered during anesthesia workup, serum cholinesterase (ChE), as a prognostic factor for patients with radiation-recurrent prostate cancer undergoing salvage radical prostatectomy. ChE modulates cellular proliferation and differentiation, and ChE levels are decreased in patients with cancer [59,60]. While pre-RP serum PSA is the strongest predictor of BFS and OS, as guidelines such as the EAU criteria for salvage RP emerge for stratifying patients toward intervention, Vartolomei et al. sought to analyze preoperative ChE as an additional predictor for outcomes. The authors found that decreased pre-RP serum ChE levels < 5 kU/L were associated with metastasis to lymph nodes (*p* = 0.004) and were an independent predictor of OS (HR: 0.68, 95%CI: 0.48–0.96, *p* = 0.03), although they did not predict BFS [61]. This highlights the value of ChE as a biomarker of micrometastasis, but further research is needed to assess this relationship, especially since ChE is nonspecific to prostate cancer.

#### 3.3.4. Comparison to Alternate Salvage Therapy

Compared to salvage focal therapy, salvage radical prostatectomy treats the entire gland, which may improve the prognostic value of post-salvage RP PSA screening. Salvage RP also affords the ability to treat cancer recurrence within the seminal vesicles, which may not be possible using other salvage modalities. Whereas brachytherapy seeds or EBRT fiducial markers may complicate salvage HIFU, salvage RP can also be performed in patients who have previously undergone either EBRT or brachytherapy. Additionally, salvage surgery offers the possibility of performing lymph node dissection, which may aid in restaging. Still, the procedure is extremely technically demanding, and intraoperative and postoperative complications should not be understated. Furthermore, compared to other salvage modalities such as HIFU or cryoablation, salvage RP may increase the risk of incontinence and impotence [62].

### 3.4. Future Directions and Ongoing Clinical Trials

The results of the abovementioned studies underscore the clinical utility of salvage radical prostatectomy in patients with radiation-resistant prostate cancer. Nevertheless, there is still a need to improve outcomes and optimize the therapeutic approach in patients with radio-recurrent disease. Despite the demonstrable safety and efficacy of post-radiation salvage RP, important questions remain unanswered. Perhaps most importantly, there are no prospective, randomized control trials evaluating the efficacy or toxicity of salvage RP after radiation compared to other modalities such as HIFU or cryoablation. Furthermore, it is unknown whether the type of primary radiotherapy (i.e., EBRT, brachytherapy, or a combination) impacts functional or oncologic outcomes following salvage RP.

While there is a plethora of ongoing clinical trials in the setting of biochemically recurrent prostate cancer, most are focused on improving the detection of recurrent disease or on novel treatment modalities, and there is a relative paucity of current trials specifically examining post-radiation salvage RP. Still, there are several current clinical trials that include patients undergoing post-radiation salvage RP, which will hopefully add to the understanding of salvage surgery after radiation. The Trace-II trial (NCT05555017) is a phase II trial that seeks to assess the feasibility of PSMA-bound radiotracers to guide salvage surgery in patients with BCR. The trial is not yet recruiting but will include patients with local recurrence who have previously undergone primary treatment with either RT or RP, and therefore may represent an alternative to salvage RP. Functional and oncologic outcomes from trials such as this will be key in determining the utility and role of PSMA-radioguided salvage surgery [63]. The VA STARPORT trial (NCT04787744) is a combined phase II/III that is actively recruiting and seeks to determine if adding PET-directed local therapy improves disease control compared to standard systemic therapy alone in patients with oligorecurrent prostate cancer after primary treatment. This trial will include patients with post-radiation local recurrence treated with salvage RP and, thus, will give additional insight into functional and oncologic outcomes in this population [64]. Lastly, NCT00791115 is a small clinical trial that seeks to enroll 16 patients with biopsy-proven recurrence after RT who will undergo salvage RP with the intent to validate virtual dosimetry plans for tumor-targeted salvage brachytherapy. The secondary outcomes include the evaluation of surgical margin status, toxicity, and biochemical control after salvage RP [65]. While these trials will ideally add to the understanding of post-radiation salvage RP, a dedicated clinical trial focusing primarily on this patient population remains to be seen.

## 4. Conclusions

Salvage radical prostatectomy remains an effective but underutilized therapeutic option for men with localized prostate cancer recurrence after radiotherapy. While it offers long-term oncologic and survival benefits, salvage RP after RT is a technically difficult procedure due to obliterated tissue planes and fibrotic reaction. Although functional outcomes have improved over time, post-radiation salvage RP still poses an elevated risk of erectile dysfunction and urinary incontinence. Given these risks, patient selection is paramount in determining when to offer salvage RP, a recommendation that has found its way into international society guidelines. Patients who are likely to benefit most from salvage RP include those with long life expectancy who have lower preoperative PSA, a lower Gleason score, and lower post-prostatectomy staging and who do not have nodal involvement. As the detection of radio-recurrent disease continues to improve with novel imaging techniques, decision making surrounding salvage therapy will continue to become more individualized, and oncologic outcomes will similarly become more promising.
